# Counteracting Angiotensinogen Small-Interfering RNA-Mediated Antihypertensive Effects With REVERSIR

**DOI:** 10.1161/HYPERTENSIONAHA.124.22878

**Published:** 2024-05-01

**Authors:** Dien Ye, Edwyn O. Cruz-López, Richard van Veghel, Ingrid M. Garrelds, Anne Kasper, Kelly Wassarman, Ho-Chou Tu, Ivan Zlatev, A.H. Jan Danser

**Affiliations:** Division of Pharmacology and Vascular Medicine, Department of Internal Medicine, Erasmus MC, University Medical Center Rotterdam, the Netherlands (D.Y., E.O.C.-L., R.v.V., I.M.G., A.H.J.D.).; Alnylam Pharmaceuticals, Cambridge, MA (A.K., K.W., H.-C.T., I.Z.).

**Keywords:** angiotensinogen, blood pressure, hypertension, renin-angiotensin system, RNA

## Abstract

**BACKGROUND:**

Small-interfering RNA (siRNA) targeting hepatic AGT (angiotensinogen) mRNA depletes AGT, lowering blood pressure for up to 6 months. However, certain situations may require a rapid angiotensin increase. The REVERSIR (RVR) - reverse siRNA silencing technology a potential approach to counteract siRNA effects.

**METHODS:**

Spontaneously hypertensive rats received 10 mg/kg AGT siRNA, and 3 weeks later were given AGT-RVR (1, 10, or 20 mg/kg). One week after AGT-RVR dosing, a redose of AGT siRNA assessed its post-AGT-RVR effectiveness for 2 weeks. Additionally, the impact of AGT-RVR after an equihypotensive dose of valsartan (4 mg/kg per day) was examined.

**RESULTS:**

Baseline mean arterial pressure (MAP) was 144±1 mm Hg. AGT siRNA reduced MAP by ≈16 mm Hg and AGT by >95%, while renin increased 25-fold. All AGT-RVR doses restored MAP to baseline within 4 to 7 days. Notably, 10 and 20 mg/kg restored AGT and renin to baseline, while 1 mg/kg allowed ≈50% AGT restoration, with renin remaining above baseline. A second AGT siRNA treatment, following 1 mg/kg AGT-RVR, reduced MAP to the same degree as the initial dose, while following 10 mg/kg AGT-RVR, it resulted in ≈50% of the first dose’s MAP effect at 2 weeks. The valsartan-induced MAP reduction was unaffected by AGT-RVR.

**CONCLUSIONS:**

In spontaneously hypertensive rats, angiotensinogen-RVR dose-dependently reversed AGT siRNA-induced AGT reduction, normalizing MAP. MAP normalization persisted even with 50% recovered AGT levels, likely due to upregulated renin maintaining adequate angiotensin generation. Post-AGT-RVR dosing, a second AGT siRNA dose lowered MAP again.

**NOVELTY AND RELEVANCE**

**What Is New?**

Small-interfering RNA (siRNA) targeting hepatic AGT (angiotensinogen) mRNA depletes AGT, lowering blood pressure for up to 6 months. This study shows that the reverse siRNA silencing (RVR) technology is capable of fully counteracting siRNA-induced effects.

**What Is Relevant**

In patients treated with AGT siRNA, RVR is a highly selective tool that can be applied to reverse its effects in situations that may require a rapid angiotensin increase.

**Clinical/Pathophysiological Implications?**

Renin-angiotensin system activation, if needed during pregnancy, shock, or emergency surgery in patients treated with AGT siRNA, is now feasible, not only by acutely infusing angiotensin II, but also by applying RVR to reverse the AGT siRNA-mediated AGT suppression.

Blood pressure management involves antihypertensive therapies blocking the renin-angiotensin system (RAS). Yet, such therapies might be inadequate due to poor patient adherence, with daily oral therapies, and the so-called RAS escape phenomenon, elicited by the compensatory renin elevation upon RAS blockade.^1^ Recently, evidence points toward targeting hepatic AGT (angiotensinogen) as a novel approach to block the RAS pathway that could circumvent the RAS escape phenomenon.^2^ Such blockade can be achieved by using a GalNAc (*N*-acetylgalactosamine)-conjugated small-interfering RNA (siRNA) targeting liver *Agt* mRNA.^3,4^ This approach is based on the fact that the asialoglycoprotein receptor is expressed on hepatocytes and can be harnessed for delivering GalNAc-siRNAs inside hepatocytes via clathrin-mediated endocytosis.^5^ Advancements in chemical modification patterns have led to enhanced stabilization chemistry in siRNA designs, resulting in significantly improved potency and duration.^6–8^ Indeed, it is shown that target gene silencing by GalNAc-siRNAs can persist for several months after a single-dose administration in humans.^3,9^

Preclinical data now support that AGT siRNA lowers blood pressure and exerts cardio- and renoprotection in a variety of animal models with hypertension and chronic kidney damage.^4,10–12^ Interestingly, these studies simultaneously showed that Ang (angiotensin) generation in the heart, brain, adipose tissue, and kidney relied on hepatic AGT and that the massive renin rises accompanying near-complete (>99%) AGT lowering tended to further consume any remaining AGT, resulting in the virtual disappearance of Ang II (angiotensin II). Moreover, a phase 1 study (URL: https://www.clinicaltrials.gov; Unique identifier: NCT03934307) in patients with hypertension revealed that zilebesiran, an investigational subcutaneously administered GalNAc-siRNA therapeutic targeting hepatic AGT, not only lowered serum AGT in a dose-dependent manner (>90% at a dose of 800 mg) but also reduced blood pressure at single doses of ≥200 mg for up to 24 weeks.^3^ Single doses of zilebesiran were well tolerated, with no observed hypotension, hyperkalemia, or worsening renal function.

A potential benefit of AGT siRNA over currently available antihypertensive therapies is better treatment adherence due to the low frequency at which siRNA needs to be dosed. However, RAS activation may be necessary to maintain adequate arterial pressure and tissue perfusion during pregnancy or certain hypotensive medical scenarios, such as shock or emergency surgery. In rodent models, AGT siRNA-mediated blood pressure lowering could be rapidly reversed by administration of Ang II or norepinephrine, or gradually reversed by fludrocortisone or high salt intake.^13^ Recently, the REVERSIR (RVR) - reverse siRNA silencing technology was reported as a potential approach to counteract siRNA-mediated pharmacodynamic effects.^14^ This technology involves short, synthetic single-stranded oligonucleotides that are complementary to the siRNA guide (antisense) strand loaded in the RNA-induced silencing complex and that target hepatocytes using the same GalNAc delivery approach. Upon binding with high affinity to the RNA-induced silencing complex-loaded siRNA guide strand, the RVR molecule blocks recognition and silencing of the corresponding mRNA and, therefore, allows for subsequent resumption in protein production.

The aim of the present study was to evaluate the use of AGT-RVR, a siRNA silencing, RVR molecule specifically designed for the rat AGT siRNA molecule used in this study, to reverse the potent AGT- lowering effect and blood pressure–lowering effect of AGT siRNA in spontaneously hypertensive rats (SHR). As a comparator, we used the AT_1_ (Ang II type 1) receptor blocker valsartan, which lowers blood pressure in SHR to the same degree as AGT siRNA.^4^ Importantly, the renin rise accompanying valsartan treatment is often accompanied by a modest drop in circulating AGT. Finally, to investigate a potential clinical situation where a patient, after having recovered, would require redosing of AGT siRNA, we also evaluated the efficacy of a second AGT siRNA dose after AGT-RVR dosing.

## METHODS

### Data Availability

All supporting data are available within the article and in the Supplemental Material.

### Animal Studies

**Figure 1.**
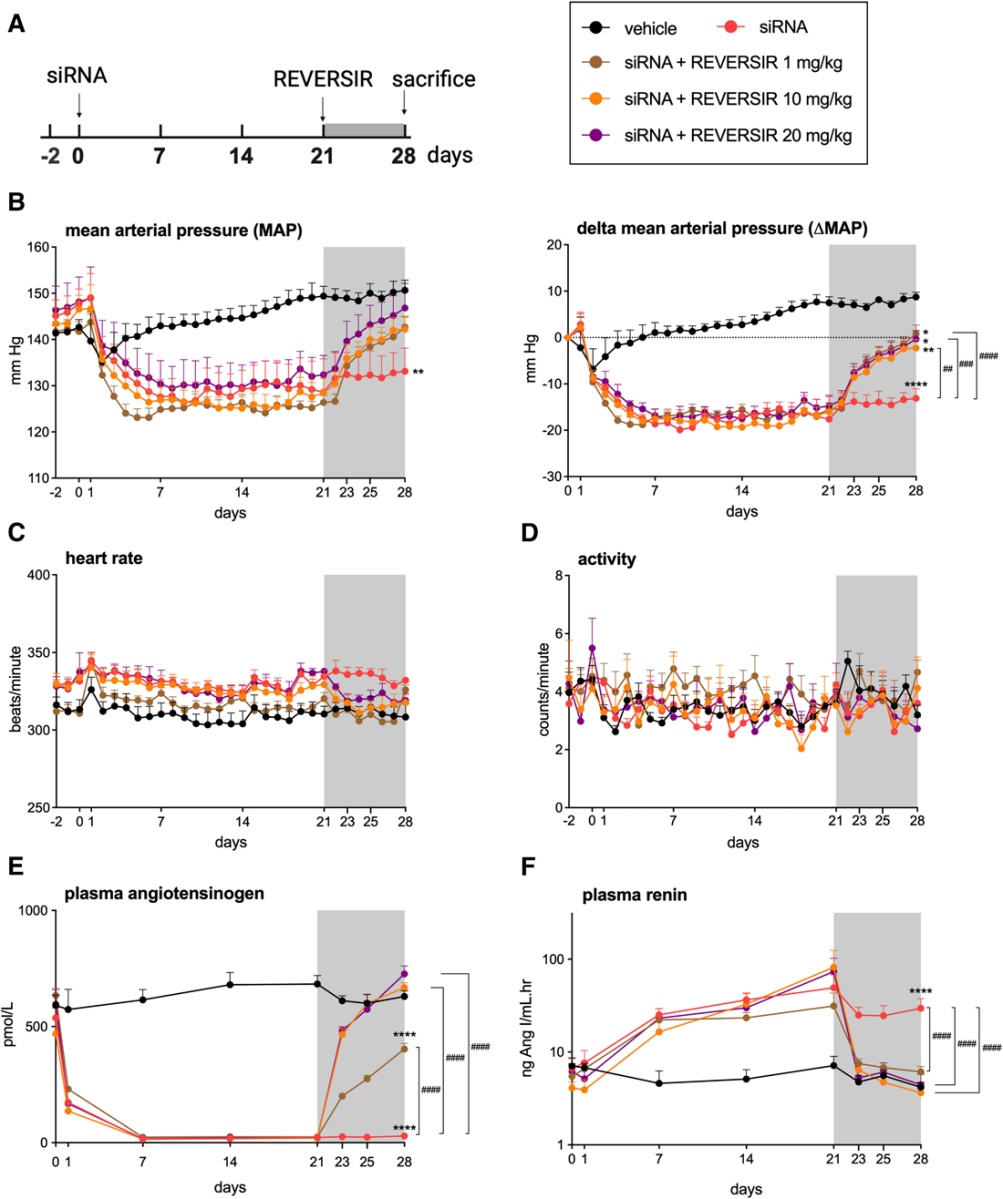
**AGT (angiotensinogen)-RVR reverses the antihypertensive and AGT-lowering effect of AGT small-interfering RNA (siRNA) in spontaneously hypertensive rats. A**, Experimental set up. **B**, Mean arterial pressure (MAP) and delta MAP. **C**, Heart rate. **D**, Activity. **E**, Plasma AGT. **F**, Plasma renin. Data are mean±SEM of n=6–7. REVERSIR (RVR) indicates reverse siRNA silencing. *P<0.05, **P<0.01, ****P<0.0001 vs vehicle; ##*P*<0.01, ###*P*<0.001, ####*P*<0.0001 vs indicated group.

**Figure 2.**
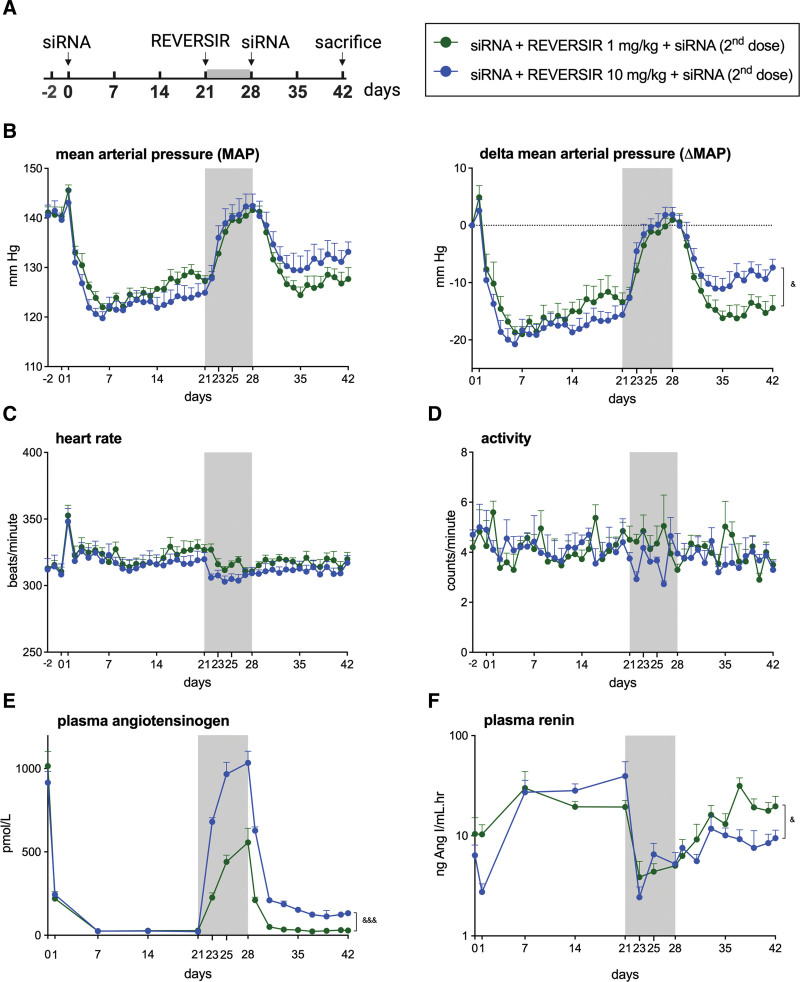
**Reintroducing AGT (angiotensinogen) small-interfering RNA (siRNA) after AGT-RVR lowers blood pressure and AGT again in spontaneously hypertensive rats. A**, Experimental set up. **B**, Mean arterial pressure (MAP), and delta MAP. **C**, Heart rate. **D**, Activity. **E**, Plasma AGT. **F**, Plasma renin. Data are mean±SEM of n=4. REVERSIR (RVR) indicates reverse siRNA silencing. &*P*<0.05, &&&*P*<0.001 vs indicated group.

All animal experiments were performed under the regulation and approval of the Animal Care Committee of the Erasmus MC (protocol No. 2115216). Male, 10-week-old male SHR (weight, 290–300 g) were purchased from Janvier, Le Genest-Saint Isle, France. Rats were maintained on a 12-hour light/dark cycle with access to standard rat chow food and water ad libitum. At the age of 12 weeks, rats were treated with either vehicle (n=7), AGT siRNA (10 mg/kg by subcutaneous injection, n=32), or valsartan (4 mg/kg per day by the osmotic mini pump; n=16; Novartis, Arnhem, the Netherlands) for 3 weeks. Minipumps (Alzet, model 2ML4) were from Durect Corporation, Cupertino, CA. After 3 weeks, in 18 of the 32 AGT siRNA-treated rats, AGT-RVR was injected subcutaneously at 3 different doses (1, 10, and 20 mg/kg, n=6 for each; Figure 1A), and then the rats were monitored for ≥1 week, after which they were sacrificed. Similarly, after 3 weeks, in 8 of the 16 valsartan-treated rats, AGT-RVR was injected subcutaneously at a dose of 10 mg/kg (Figure S1A) and then these rats were also monitored for ≥1 week, after which they were sacrificed. The 7 vehicle-treated rats were sacrificed after 4 weeks, and this was also the case for 6 AGT siRNA-treated rats and 8 valsartan-treated rats that did not receive AGT-RVR. Finally, after 3 weeks, 8 of the AGT siRNA-treated rats were injected subcutaneously with AGT-RVR at 2 different doses (1 or 10 mg/kg, n=4 for each; Figure 2A), monitored for 1 week, and then injected again subcutaneously with AGT siRNA (10 mg/kg) and followed for ≥2 weeks, after which they were sacrificed. Blood pressure, heart rate, and activity were measured by radiotelemetry transmitters (HD-S10, Data Sciences International, St. Paul, MN), implanted 2 weeks before the start of treatment. Inhaled anesthesia with isoflurane was administered during radiotelemetry transmitter and minipump implantation. Blood samples for the measurement of renin and AGT were collected at regular time points by venipuncture from the lateral tail vein. At the end of the 4- or 6-week treatment period, rats were anesthetized by inhalation of isoflurane and exsanguinated. Blood was collected in EDTA tubes (MiniCollect 1 mL K3EDTA, Greiner Bio-One GmBh, Kremsmünster, Austria), and plasma was obtained after centrifugation at 4 °C for 10 minutes. Kidneys and liver were rapidly excised, weighed, and divided into transverse segments that were snap-frozen in liquid nitrogen.

### Oligonucleotide Synthesis

The AGT siRNA consisted of a chemically modified antisense strand hybridized with a chemically modified sense strand as previously reported.^7^ Oligonucleotides for AGT siRNA were synthesized as described previously.^4^ To ensure selective and efficient delivery to hepatocytes, GalNAc, a high-affinity ligand for the hepatocyte-specific asialoglycoprotein receptor was attached to the 3′ end of the sense strand.^5^ AGT-RVR was synthesized on a MerMade-12 DNA/RNA synthesizer. Sterling solvents/reagents from Glen Research, 2′-deoxy nucleoside phosphoramidites from Thermo, and 2′-OMe nucleoside phosphoramidites from Hongene were all used as received. GalNAc controlled pore glass support was prepared and used as previously described.^5^ Low-water content acetonitrile was purchased from EMD Chemicals (Merck KGaA, Darmstadt, Germany). A solution of 0.6 mol/L 5-(S-ethylthio)-1H-tetrazole in acetonitrile was used as the activator. The phosphoramidite solutions were 0.15 mol/L in anhydrous acetonitrile with 15% dimethylformamide as a co-solvent for 2′-OMe uridine and cytidine. The oxidizing reagent was 0.02 mol/L I_2_ in tetrahydrofuran/pyridine/water. N,N-dimethyl-N′-(3-thioxo-3H-1,2,4-dithiazol-5-yl)methanimidamide, 0.09 mol/L in pyridine, was used as the sulfurizing reagent. The detritylation reagent was 3% dichloroacetic acid in dichloromethane. After completion of the solid-phase synthesis, the controlled pore glass solid support was washed with 5% (v/v) piperidine in anhydrous acetonitrile 3× with 5-minute holds after each flow. The support was then washed with anhydrous acetonitrile and dried with argon. The oligonucleotides were then incubated with 28% to 30% (w/v) NH_4_OH, at 35 °C for 20 hours. The solvent was collected by filtration, and the support was rinsed with water before analysis and subsequent high-performance liquid chromatography purification of the AGT-RVR.

### Biochemical Measurements

The plasma concentrations of AGT and renin were measured by enzyme-kinetic assay as described before.^4,15^ The lower limits of detection of the AGT and renin assays were 0.2 pmol/mL and 0.17 ng Ang I/mL per hour, respectively. In the case that measurements were at or below the lower limit of detection, this limit was applied to allow for statistical analysis.

### Quantitative Polymerase Chain Reaction

Total RNA was isolated from snap-frozen liver using the TRI reagent (Sigma-Aldrich), purified by using Direct-zol RNA Miniprep Kits (Zymo Research Corporation, Irvine, CA), and reverse transcribed into cDNA using the Maxima H Minus First Strand cDNA Synthesis Kit (Thermo Scientific, Venlo, the Netherlands). cDNA was amplified in triplicate in 40 cycles (denaturation at 95 °C for 3 minutes, thermal cycling at 95 °C for 3 seconds, annealing/extension at 60 °C for 20 seconds) followed by a melt curve with a CFX384 (Bio-Rad, Veenendaal, the Netherlands) using PowerTrack SYBR Green Master Mix (Thermo Scientific). Intron-spanning oligonucleotide primers were designed with NCBI, AGT (forward, CCAGCACGACTTCCTGACTT; reverse, GCAGGTTGTAGGATCCCCGA), β-actin (forward, GGGAAATCGTGCGTGACATT; reverse, GCGGCAGTGGCCATCTC), and B2M (β2-microglobulin; forward, ATGGCTCGCTCGGTGACCG; reverse, TGGGGAGTTTTCTGAAT GGCAAGCA). The ΔΔCt method was used for relative quantification of mRNA expression levels, using the housekeeping gene B2M and β-actin for normalization.

### Western Blotting

Snap-frozen liver and kidneys of rats were homogenized on ice in a buffer containing 0.3 mol/L sucrose, 50 mmol/L Tris-HCl pH 7.5, 1 mmol/L EDTA, 1 mmol/L EGTA, 50 mmol/L sodium fluoride, 1 mmol/L DTT, and 1 mmol/L PMSF supplemented with protease inhibitors. Subsequently, ≈25 μg of protein (DC protein assay kit; Bio-Rad) was separated by electrophoresis on a Criterion TGX precast protein gel (Bio-Rad) and transferred to a membrane using the Trans-Blot Turbo Transfer System (Bio-Rad). Membranes were blocked with 5% BSA in Tris-buffered saline containing 0.1% Tween-20, followed by incubation overnight at 4 °C with a primary antibody directed against AGT (1:100; Immuno-Biological Laboratories, Fujioka, Japan). The specificity of this antibody had been validated before, by demonstrating a band at ≈53 kDa in tissues obtained from wild-type mice but not in tissues from hepAGT^−/−^ mice.^13^ After washing, blots were incubated with an antirabbit horseradish peroxidase-conjugated secondary antibody (1:3000; Bio-Rad). Signals were detected by chemiluminescence (Clarity Western ECL substrate; Bio-Rad) and quantified using ImageQuant LAS 4000 (GE Healthcare, Diegem, Belgium). GADPH (glyceraldehyde-3-phosphate dehydrogenase; 1:5000; GeneTex, Irvine, CA) was used for the normalization of protein levels.

### Statistics

Data are expressed as mean±SEM in case of normal distribution and median with interquartile range in case of non-normal distribution. Statistical analysis was undertaken only for studies where each group size was at least n=6. Nonnormally distributed data were log-transformed before statistical analysis. Data were analyzed by 1-way ANOVA and mixed linear models, using treatment and time as fixed effects. If significant (*F* in ANOVA *P*<0.05), selected post hoc analyses (Tukey test, when among all groups; Dunnett test, when comparing groups to control) were performed. When comparing 2 groups, an unpaired *t* test was performed. All analyses were performed using Prism, version 9.0.0 (GraphPad Software Inc, La Jolla, CA).

## RESULTS

### AGT-RVR Restores Mean Arterial Pressure and AGT in AGT siRNA-Treated But Not in Valsartan-Treated SHR

In agreement with a previous study in SHR, 10 mg/kg AGT siRNA lowered mean arterial pressure (MAP) by ≈20 mm Hg and lowered circulating AGT by >95% (Figure 1B and 1E). Renin levels rose ≈25-fold (Figure 1F). Maximum changes were achieved after 1 week of siRNA treatment and remained consistent until the animals were sacrificed at 4 weeks. AGT siRNA did not affect heart rate or activity (Figure 1C and 1D). Administering AGT-RVR after 3 weeks of AGT siRNA treatment reversed the blood pressure–lowering effect within 5 to 7 days. This reversal occurred uniformly across all tested AGT-RVR doses (1, 10, and 20 mg/kg). In contrast, 10 and 20 mg/kg AGT-RVR fully restored circulating AGT and renin, while 1 mg/kg AGT-RVR partially restored circulating AGT and maintained circulating renin levels numerically above those in vehicle-treated rats. The changes in circulating AGT and renin occurred within the same time frame (5–7 days) as those in MAP.

The blood pressure–lowering effects of valsartan after 4 weeks were similar to those of AGT siRNA, although initially (after 1 week of treatment) its effects tended to be stronger (Figure S1B). In parallel with these observations, the heart rate initially increased and then levelled-off again to normal (Figure S1C). No changes in activity occurred (Figure S1D). Valsartan increased circulating renin to the same degree as AGT siRNA (Figure S1F), and this renin rise resulted in a 40% to 50% drop in circulating AGT (Figure S1E). Injecting AGT-RVR after 3 weeks of valsartan did not alter this outcome.

In summary, these data demonstrate that AGT-RVR dose-dependently reversed the blood pressure and AGT-lowering effects specifically mediated by AGT siRNA and did not have effects when administered on top of valsartan.

### Second Dose of AGT siRNA Lowers MAP Following the Treatment With AGT-RVR

To evaluate whether AGT siRNA can re-exert its blood pressure–lowering effect post-AGT-RVR administration, rats initially treated with AGT siRNA, whose MAP had been restored after AGT-RVR administration at doses of either 1 or 10 mg/kg, were given a second dose of AGT siRNA. With 1 mg/kg AGT-RVR, a second dose of 10 mg/kg AGT siRNA lowered MAP within 1 week to the same extent as it did before AGT-RVR administration (Figure 2B). However, with 10 mg/kg AGT-RVR, a second dose of 10 mg/kg AGT siRNA lowered MAP within 1 week to ≈50% of the effect of the initial AGT siRNA dose (Figure 2B). No changes in heart rate and activity were observed (Figure 2C and 2D). Similar to the MAP observations, administering AGT-RVR at 10 mg/kg on top of AGT siRNA fully restored circulating AGT and renin, while at 1 mg/kg, it restored circulating AGT by ≈50%, with renin remaining slightly elevated. In this context, a second siRNA dose once again lowered circulating AGT, reaching the lowest AGT levels and highest renin levels in the rats treated with 1 mg/kg AGT-RVR (Figure 2E and 2F).

These data demonstrate that retreatment with AGT siRNA after AGT-RVR treatment is effective, with complete restoration of AGT siRNA effects when a lower dose of AGT-RVR has been administered.

### Liver and Kidney AGT Expression

**Figure 3.**
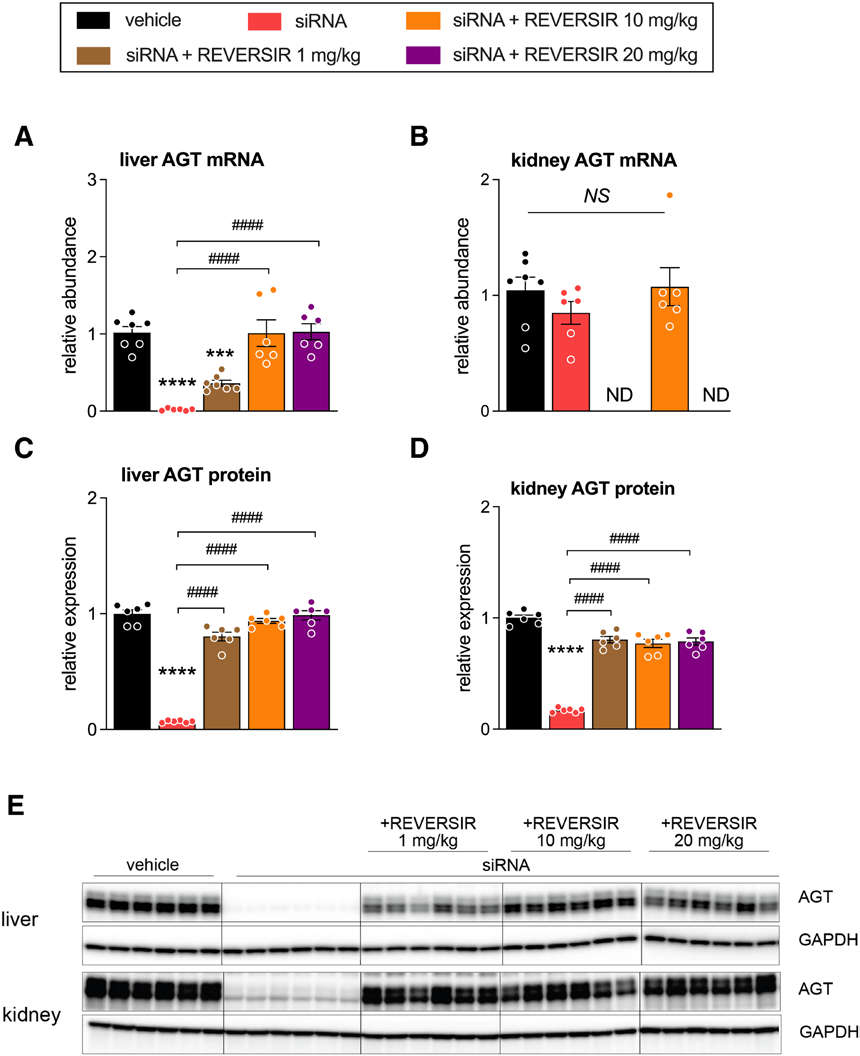
**AGT (angiotensinogen) mRNA and protein expression in liver and kidney of spontaneously hypertensive rats treated with AGT small interfering RNA (siRNA) and AGT-RVR. A**, Hepatic AGT mRNA levels (normalized vs β-actin and β2-microglobulin). **B**, Renal AGT mRNA levels. **C**, Hepatic AGT protein levels (normalized vs GADPH [glyceraldehyde-3-phosphate dehydrogenase]). **D**, Renal AGT protein levels. **E**, Western blotting images of AGT in liver and kidney. Data are mean±SEM of n=6–7. ND indicates not done; and REVERSIR (RVR) indicates reverse siRNA silencing. ***P<0.001, ****P<0.0001 vs vehicle; ^####^*P*<0.0001 vs indicated group.

In the SHRs, AGT siRNA significantly suppressed the hepatic *Agt* mRNA and AGT protein levels (Figure 3A, 3C, and 3E). It also reduced kidney AGT protein levels (Figure 3D and 3E) but did not affect kidney *Agt* mRNA (Figure 3B). Administration of AGT-RVR restored hepatic Agt mRNA, renal and hepatic AGT protein levels back to their original values in a dose-dependent manner, with a similar effect of 10 or 20 mg/kg AGT-RVR. Valsartan upregulated hepatic AGT mRNA levels (Figure S2A), without altering hepatic or renal AGT protein levels (Figure S2B through S2D).

**Figure 4.**
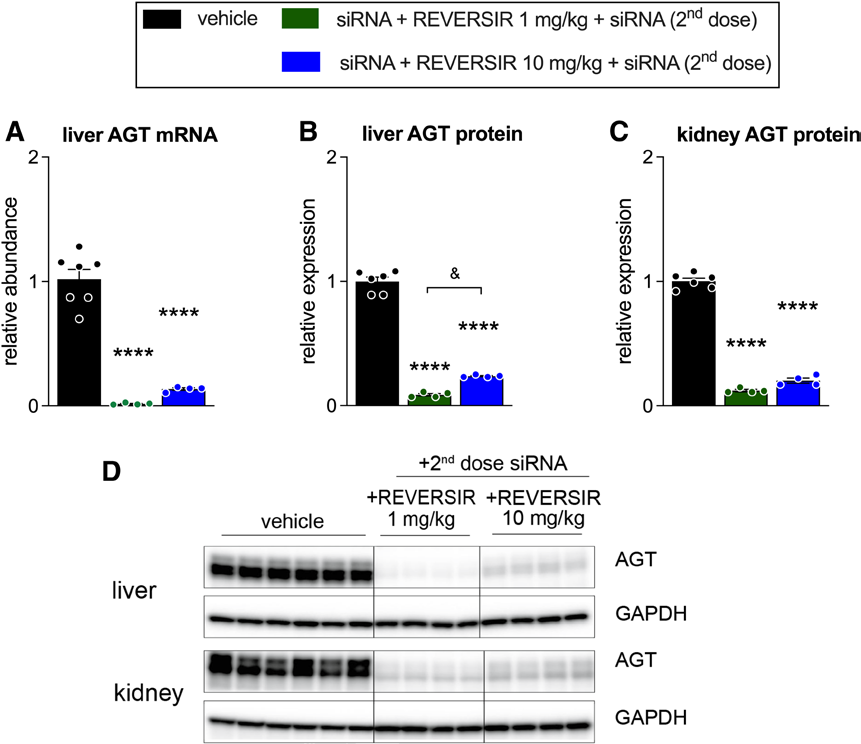
**AGT (angiotensinogen) mRNA and protein expression in the liver and kidney of spontaneously hypertensive rats receiving a second dose of AGT small interfering RNA (siRNA) after AGT-RVR. A**, Hepatic AGT mRNA levels (normalized vs β-actin and β2-microglobulin). **B**, Hepatic AGT protein levels (normalized vs GADPH [glyceraldehyde-3-phosphate dehydrogenase]). **C**, Renal AGT protein levels. **D**, Western blotting images of AGT in liver and kidney. Data are mean±SEM of n=4–7. REVERSIR (RVR) indicates reverse siRNA silencing. ****P<0.0001 vs vehicle; &*P*<0.05 indicated group.

When a second dose of AGT siRNA was administered following treatment with either 1 or 10 mg/kg AGT-RVR treatment, hepatic AGT mRNA and protein levels, as well as renal AGT protein levels were lowered again (Figure 4A through 4D versus Figure 3), with the lowest levels observed in rats treated with 1 mg/kg AGT-RVR.

These data indicate that GalNAc-conjugated AGT siRNA and AGT-RVR are liver-specific, and that renal AGT protein is liver-derived. They also show that AGT-RVR dose-dependently reverses the effect of AGT siRNA, and that applying a second AGT siRNA dose after AGT-RVR dosing can suppress hepatic AGT expression again.

## DISCUSSION

This study demonstrates that AGT-RVR can reverse the AGT- and blood pressure–lowering effects of AGT siRNA in a hypertensive rat model, the SHR. AGT-RVR did not affect the AGT- and blood pressure–lowering effect of valsartan. Finally, once AGT-RVR had restored AGT and blood pressure, reapplication of AGT siRNA on top of AGT-RVR lowered AGT and blood pressure again.

A single subcutaneous dose of AGT siRNA of 10 mg/kg lowered MAP in SHRs to the same degree as valsartan applied by osmotic minipump at a dose of 4 mg/kg per day. Maximum efficacy of AGT siRNA was reached after ≈1 week, reflecting the time required to allow siRNA to maximally reduce circulating AGT protein. At a dose of 10 mg/kg, the reduction in circulating AGT was >95%. Similar reductions were seen in the liver, both at the mRNA and protein levels. Yet, in the kidney, only AGT protein was lowered, and not AGT mRNA. The latter confirms the liver specificity of GalNAc-siRNA conjugates. The former demonstrates that, although AGT is expressed in the kidney, the main, if not the only source of AGT protein in the kidney is the liver. Indeed, in agreement with this concept, the suppression of hepatic AGT was accompanied by a reduction in renal Ang II formation, and when deleting circulating AGT >99.9%, renal Ang II virtually disappeared.^4^

AGT-RVR dose-dependently restored hepatic *Agt* mRNA levels, and a similar dose dependency was observed for the return of circulating AGT. This implies that at a dose of 1 mg/kg, hepatic *Agt* mRNA and circulating AGT were back to ≈50% of their original (pre-AGT siRNA) levels, while at doses of 10 mg and higher, they had fully recovered. As expected, renin changes opposed those in AGT: in the absence of AGT, renin rose 25-fold, while it dropped to normal again when circulating AGT normalized. Only when circulating AGT had not yet returned to its pre-AGT siRNA levels, renin levels remained moderately elevated. The latter likely explains why MAP returned to normal even at an AGT-RVR dose of 1 mg/kg, which allowed circulating AGT to recover by only 50%. The combination of a 50% reduction in AGT and a modest elevation in renin was sufficient to normalize RAS activity and MAP. The dose dependency of the changes in hepatic and renal AGT protein was less clear, most likely relating to the fact that tissue AGT levels were quantified by Western blotting, while those in the blood relied on the much more sensitive enzyme-kinetic assay.

Valsartan treatment, by interfering with the negative feedback loop that allows Ang II to suppress renin release was accompanied by renin rises. These rises were comparable to those observed during AGT siRNA treatment. Such high renin levels will rapidly consume AGT, potentially lowering circulating AGT. This occurs, for instance, in patients with heart failure, particularly during treatment with RAS blockers and diuretics (with high renin levels^16,17^), and it also occurred in the SHR of the present study, both in blood and hepatic tissue sites. However, the reductions in AGT that were observed (40%–50%) were far below the >95% reduction observed after AGT siRNA. Most likely this was due, at least in part, to the upregulation of hepatic *Agt* mRNA expression. Importantly, under these conditions, AGT-RVR had no effect. This is not surprising, given our observation that the reduction in circulating AGT in this case was not due to a suppression of hepatic *Agt* mRNA levels by AGT siRNA but rather due to a rise in renin. These data illustrate that AGT-RVR will not be anticipated to be effective in patients in whom AGT levels are low due to mechanisms other than AGT siRNA.

Finally, we tested whether retreatment with AGT siRNA after restoring MAP to normal with AGT-RVR at doses of either 1 or 10 mg/kg would lower blood pressure again. Here, it is important to note that 1 mg/kg AGT-RVR did restore MAP, while circulating AGT levels were still at ≈50% of their pre-AGT siRNA values. Under this condition, redosing AGT siRNA at 10 mg/kg (the same dose that had been applied earlier) allowed a MAP reduction to the same degree as it did with the first AGT siRNA dose. In contrast, when AGT siRNA (10 mg/kg) was redosed on top of 10 mg/kg AGT-RVR, which had allowed circulating AGT to be fully restored, the blood pressure–lowering effect was only about half that of the first dose. As previously described,^14^ this likely reflects the differences between siRNA and RVR in terms of intracellular delivery and trafficking, as well as metabolic stability and half-life in hepatocytes. Clearly, AGT-RVR is a potent tool, and its effects on blood pressure are already maximal when applying it in a dose that is <10× that of AGT siRNA. From this perspective, it is not surprising that when RVR is given at a dose of 10 mg/kg, redosing of AGT siRNA at 10 mg/kg is insufficient to fully lower MAP to the same degree as the first AGT siRNA dose. A higher dose of AGT siRNA would likely overcome this RVR effect. It is likely that the optimal ratio of siRNA, RVR, and the second dose of siRNA will be dependent on each siRNA and RVR pair in a sequence and chemistry-dependent manner.

## PERSPECTIVES

In conclusion, together with our earlier demonstration that conventional vasopressor (norepinephrine, Ang II) therapies can acutely reverse AGT siRNA-mediated blood pressure lowering in SHR,^13^ the current data now reveal that AGT-RVR is a highly selective tool to chronically reverse the AGT siRNA-mediated AGT and blood pressure–lowering effects in SHR. These findings support further evaluation of the RVR platform to counteract siRNA-mediated effects, potentially first in humanized rodents expressing human renin and AGT, and subsequently in humans.

## ARTICLE INFORMATION

### Sources of Funding

This work was partially supported by Alnylam Pharmaceuticals. E.O. Cruz-López was supported by the Mexican National Council of Science and Technology (grant no. 739513).

### Disclosures

A. Kasper, K. Wassarman, H.-C. Tu, and I. Zlatev are employees of Alnylam Pharmaceuticals. A.H. Jan Danser received a grant from Alnylam Pharmaceuticals, which has partially supported this work. The other authors report no conflicts.

## Supplementary Material


