# Supplementary material for: Counteracting Angiotensinogen Small-Interfering RNA-Mediated Antihypertensive Effects With REVERSIR
Source: Hypertension. 2024 May 1;81(7):1491–9. doi: 10.1161/HYPERTENSIONAHA.124.22878 (PMC11177597; doi:10.1161/HYPERTENSIONAHA.124.22878)
Supplement: Supplementary file 1 [file hyp-81-1491-s001.docx]

**COUNTERACTING ANGIOTENSINOGEN SMALL INTERFERING RNA-MEDIATED ANTIHYPERTENSIVE EFFECTS WITH REVERSIR**Dien Ye^1^*, Edwyn O. Cruz-López^1^*, Richard van Veghel^1^, Ingrid M. Garrelds^1^, Anne Kasper^2^, Kelly Wassarman^2^, Ho-Chou Tu^2^, Ivan Zlatev^2^, A.H. Jan Danser^1^

^1^Division of Pharmacology and Vascular Medicine, Department of Internal Medicine, Erasmus MC, University Medical Center Rotterdam, The Netherlands;

^2^Alnylam Pharmaceuticals, Cambridge, MA, USA.

*Contributed equally

**Correspondence:** Prof. dr. A. H. Jan Danser; Division of Pharmacology, room EE1418b, Erasmus MC, Wytemaweg 80, 3015 CN Rotterdam, The Netherlands (e-mail: [a.danser@erasmusmc.nl](mailto:a.danser@erasmusmc.nl))

**SUPPLEMENTAL INFORMATION**


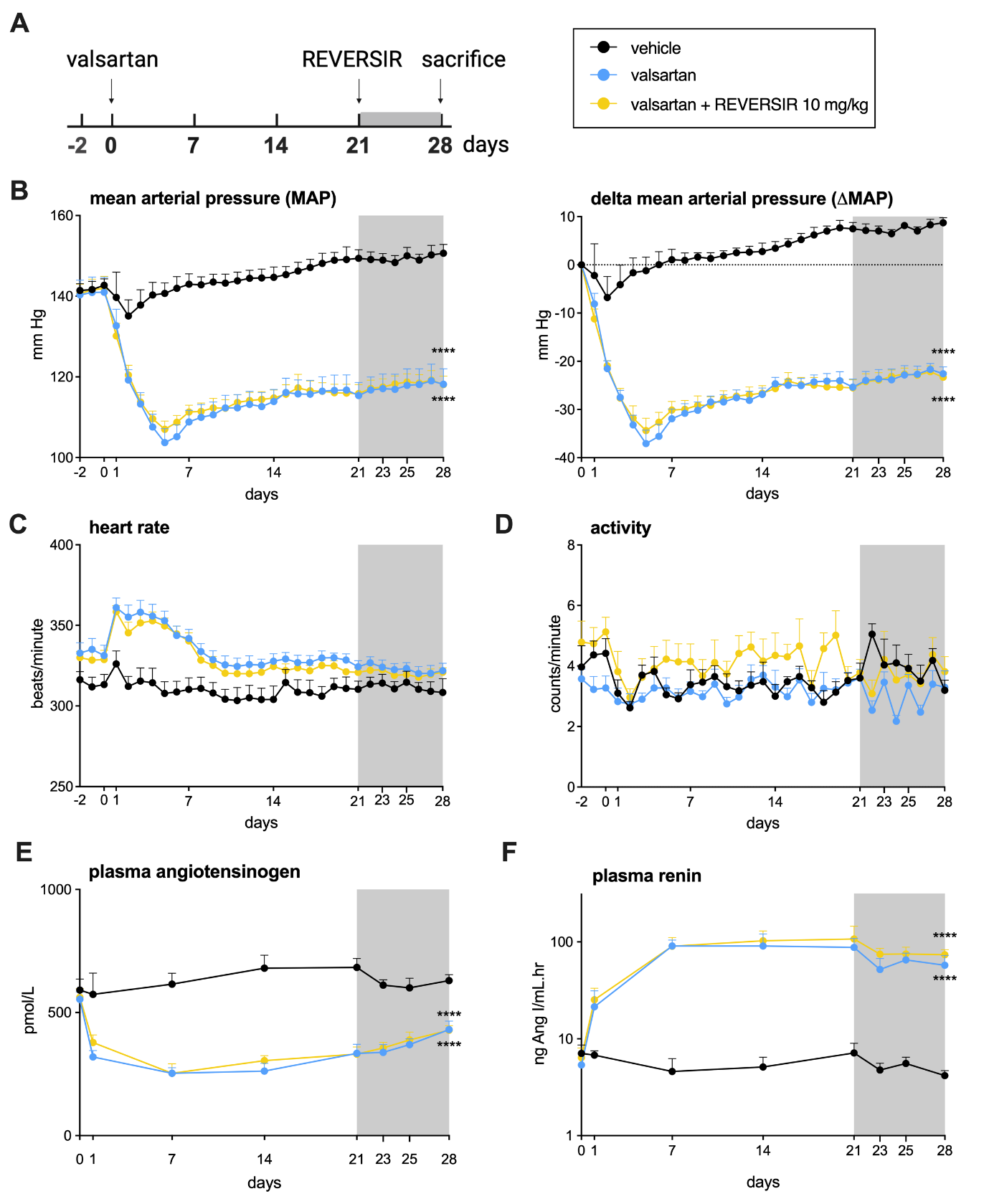


**Figure S1. Lack of effect of AGT-RVR in spontaneously hypertensive rats treated with valsartan.** (A) Experimental plan. (B) Daily mean arterial pressure and delta mean arterial pressure during valsartan with or without AGT-RVR treated. (C) Daily heart rate and (D) activity. (E) Plasma AGT and (F) renin concentration. Data are mean ± SEM of n = 7-8. *****P*<0.0001 versus vehicle.


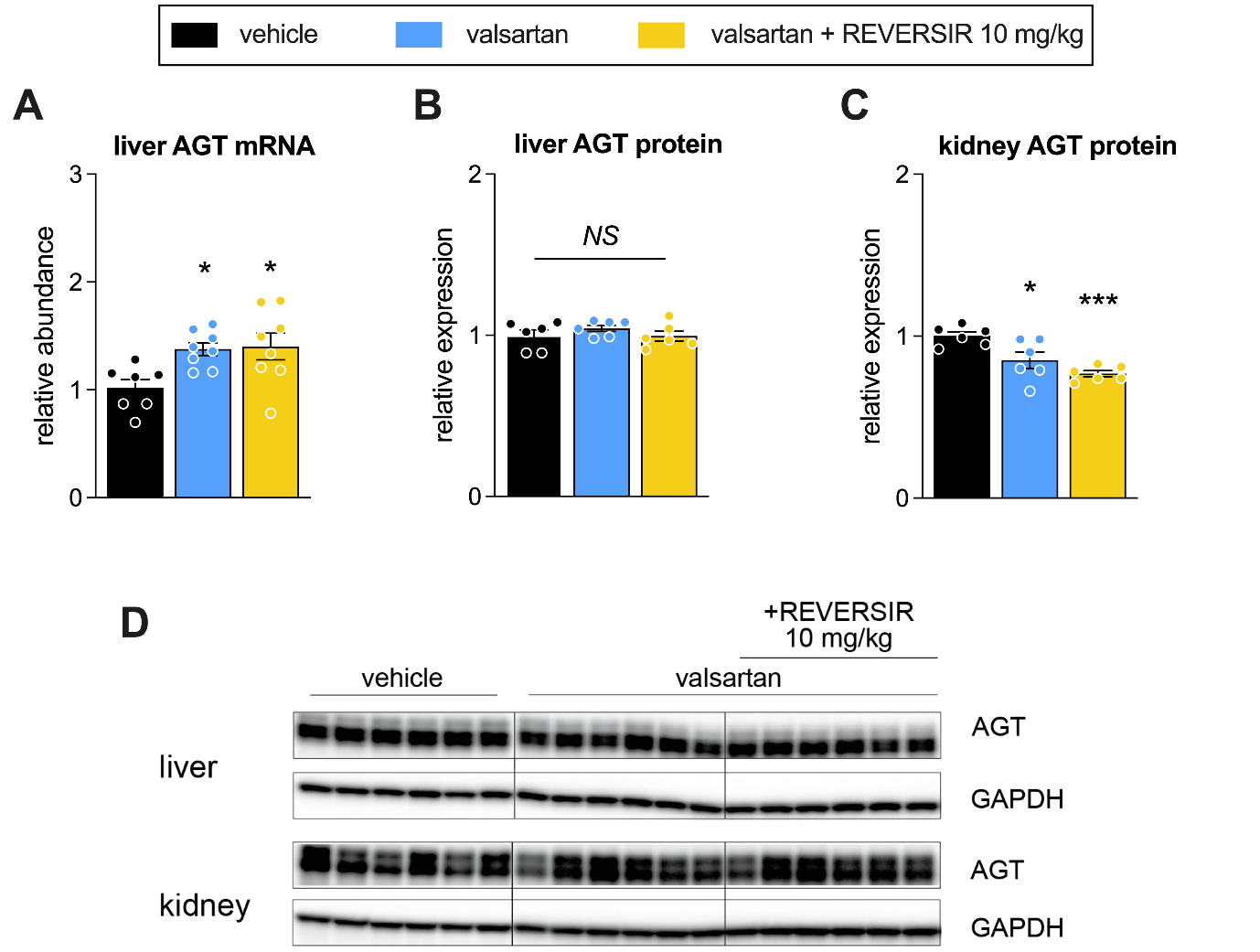


**Figure S2. Angiotensinogen (AGT) mRNA and protein expression in liver and kidney of spontaneously hypertensive rats receiving AGT-RVR on top of valsartan.** (A) Hepatic AGT mRNA levels (normalized versus β-actin and β2-microglobulin), (B) hepatic AGT protein levels (normalized versus GAPDH), (C) renal AGT protein levels, and (E) Western blotting images of AGT in liver and kidney. Data are mean ± SEM of n = 6-8. **P*<0.05, ****P*<0.001 versus vehicle.
